# A Treatise of the Diseases of Women

**Published:** 1889-02

**Authors:** 


A TREATISE OF THE DISEASES OF WOMEN, for the use of students and practitioners, by Alex. J. C. Skene, M. D., of New York, professor of Gynecology in the Long Island Medical College, Brooklyn, etc. With 251 engravings and 9 chromo-lithographs. D. Appleton & Co., 1888.

The long experience of Prof. Skene in the treatment of the diseases incident to women and the great advantages that he has enjoyed in the numerous hospitals with which he is connected, make the present handsome volume one of the best treatises now published on the subject. The book contains 950 pages printed in large clear type. It is a book of actual practice and will yield the busy practitioner the greatest returns in the shortest length of time of any work that the writer has had the pleasure of examining in some time.—B.

